# Supplementary material for: Intention to Inform Relatives, Rates of Cascade Testing, and Preference for Patient-Mediated Communication in Families Concerned with Hereditary Breast and Ovarian Cancer and Lynch Syndrome: The Swiss CASCADE Cohort
Source: Cancers (Basel). 2022 Mar 23;14(7):1636. doi: 10.3390/cancers14071636 (PMC8997156; doi:10.3390/cancers14071636)
Supplement: Supplementary file 1 [file cancers-14-01636-s001.zip › cancers-1640627-supplementary.pdf]

cancers

MDPI

Supplementary Materials

Intention to Inform Relatives, Rates of Cascade Testing, and Preference for Patient-Mediated Communication in Families Concerned with Hereditary Breast and Ovarian Cancer and Lynch Syndrome: The Swiss CASCADE Cohort

Mahesh Sarki [1], Chang Ming [1], Souria Aissaoui [2,3], Nicole Bürki [4], Maria Caiata-Zufferey [5], Tobias Ephraim Erlanger [6], Rossella Graffeo-Galbiati [7], Karl Heinimann [8,9], Viola Heinzelmann-Schwarz [4], Christian Monnerat [10], Nicole Probst-Hensch [11], Manuela Rabaglio [12], Ursina Zürrer-Härdi [13], Pierre Olivier Chappuis [14,15] and Maria C. Katapodi [1,*] and on behalf of the CASCADE Consortium†

1 Department of Clinical Research, University of Basel, 4055 Basel, Switzerland; mahesh.sarki@unibas.ch (M.S.); chang.ming@unibas.ch (C.M.)
2 Breast Center, Cantonal Hospital Fribourg, 1752 Fribourg, Switzerland; souria.aissaoui@genesupport.ch
3 GENESUPPORT, The Breast Centre, Hirslanden Clinique de Grangettes, 1224 Geneva, Switzerland
4 Women's Clinic, University Hospital Basel, 4031 Basel, Switzerland; nicole.buerki@usb.ch (N.B.); viola.heinzelmann@usb.ch (V.H.-S.)
5 Department of Business Economics, Health and Social Care, University of Applied Sciences and Arts of Southern Switzerland, 6928 Manno, Switzerland; maria.caiata@supsi.ch
6 Clinical Trials Unit, University Hospital Basel, 4031 Basel; mail@tobiaserlanger.ch
7 Oncology Institute of Southern Switzerland, EOC, 6500 Bellinzona, Switzerland; rossella.graffeogalbiati@eoc.ch
8 Institute for Medical Genetics and Pathology, University Hospital Basel, 4031 Basel, Switzerland; karl.heinimann@usb.ch
9 Research Group Human Genomics, Department of Biomedicine, University of Basel, 4031 Basel, Switzerland
10 Department of Medical Oncology, Hospital of Jura, 2800 Delemont, Switzerland; christian.monnerat@h-ju.ch
11 Swiss Tropical and Public Health Institute, University of Basel, 4123 Allschwil, Switzerland; nicole.probst@swisstph.ch
12 Department of Medical Oncology, Inselspital, Bern University Hospital, 3010 Bern, Switzerland; manuela.rabaglio@insel.ch
13 Department of Medical Oncology, Cantonal Hospital Winterthur, 8400 Winterthur, Switzerland; ursina.zuerrer@ksw.ch
14 Unit of Oncogenetics, Division of Oncology, University Hospitals of Geneva, 1205 Geneva, Switzerland; pierre.chappuis@hcuge.ch
15 Division of Genetic Medicine, University Hospitals of Geneva, 1205 Geneva, Switzerland
* Correspondence: maria.katapodi@unibas.ch; Tel.: +41-61-207-04-30
† Membership of the CASCADE Consortium is provided in the Acknowledgments.

Table S1. List of predictors selected based on the Theory of Planned Behavior (TPB) and tested in univariate logistic models.

Predictors		HBOC		LS	
		**OR**	**95% CI**	OR	95% CI
Demographic characteristics	Age	**1.01**	**1.00–1.03**	1.02	0.99–1.05
	Female (ref: male)	1.75	0.99–3.09	1.00	0.41–2.44
	High school graduate (ref: <12 years of education)	0.96	0.46–2.03	0.88	0.29–2.74
	University degree or higher (ref: <12 years of education)	0.71	0.33–1.53	0.74	0.20–2.71
	Employment (ref: no employment)	0.89	0.57–1.39	0.57	0.23–1.44
	Index case (ref: relatives)	**1.71**	**1.07–2.73**	1.57	0.52–4.72
Clinical characteristics	Carrying a pathogenic variant (ref: no variant or untested)	**2.87**	**1.71–4.81**	**4.23**	**1.15–15.63**

	Year since cancer diagnosis ≤ 5 years (ref: no cancer)	**2.16**	**1.07–4.39**	2.19	0.61–7.95
	Year since cancer diagnosis > 5 years (ref: no cancer)	1.36	0.84–2.21	1.56	0.60–4.04
	Year since genetic testing ≤ 5 years (ref: not tested)	**2.96**	**1.60–5.45**	2.85	0.62–13.12
	Year since genetic testing > 5 years (ref: not tested)	**2.41**	**1.31–4.42**	1.97	0.44–8.81
Knowledge and attitudes	Perceived cancer risk	1.01	0.89–1.15	1.06	0.85–1.31
	Genetic affinity	**1.23**	**1.03–1.49**	1.01	0.67–1.51
Subjective norms	Having a routine source of care (ref: >2 healthcare professionals)	1.59	0.47–5.33	1.19	0.12–11.54
	Healthcare provider seen most often is specialist (ref: generalist)	1.28	0.82–1.98	1.19	0.47–2.99
	Out of pocket cost is a barrier to access care	1.01	0.71–1.45	0.87	0.50–1–50
Family environment	Married or living as married (ref: single or living alone)	1.36	0.82–2.26	1.89	0.77–4.60
	Family Support in Illness	**1.29**	**1.00–1.66**	1.07	0.75–1.54

Bold = significant two-tailed *p* value ≤0.05.
